# EDTA-Modified 17β-Estradiol-Laden Upconversion Nanocomposite for Bone-Targeted Hormone Replacement Therapy for Osteoporosis

**DOI:** 10.7150/thno.37599

**Published:** 2020-02-10

**Authors:** Xiaoting Chen, Xingjun Zhu, Yan Hu, Wei Yuan, Xiaochen Qiu, Tianyuan Jiang, Chao Xia, Liqin Xiong, Fuyou Li, Yanhong Gao

**Affiliations:** 1 Department of Geriatrics, Xinhua Hospital of Shanghai Jiaotong University, School of Medicine, Shanghai 200092, China.; 2 Department of Chemistry, Fudan University, 220 Handan Road, Shanghai 200433, P.R. China.; 3 Shanghai Med-X Engineering Center for Medical Equipment and Technology, School of Biomedical Engineering, Shanghai JiaoTong University, Shanghai 200030, P. R. China

**Keywords:** osteoporosis, hormone therapy, upconversion nanoparticles, ethylenediaminetetraacetic acid

## Abstract

Hormone therapy (HT) is one of the most effective treatments for osteoporosis. However, the nonselective accumulation of hormone in organs such as breast, heart and uterus other than bones causes serious side effects, which impedes the application of HT. Hence, it is critically important to develop a HT strategy with reduced non-specific enrichment of hormone drugs in non-target tissues and enhanced bone-targeting ability.

**Methods**: Herein, a 17β-estradiol (E_2_)-laden mesoporous silica-coated upconversion nanoparticle with a surface modification of ethylenediaminetetraacetic acid (EDTA) (NaLuF_4_:Yb,Tm@NaLuF_4_@mSiO_2_-EDTA-E_2_, E_2_-csUCNP@MSN-EDTA) is developed for bone-targeted osteoporosis hormone therapy. EDTA was attached onto the surface of E_2_ upconversion nanocomposite to enhance its affinity and efficiency targeting bone tissue and cells to optimize hormone replacement therapy for osteoporosis. We characterized the size, cytotoxicity, loading and release efficiency, *in situ* and *ex vivo* imaging. Further, *in vitro* and *in vivo* osteogenic ability was tested using preosteoblast and ovariectomy mouse model of osteoporosis.

**Results**: The upconversion core of E_2_-csUCNP@MSN-EDTA nanoparticle serves as an excellent imaging agent for tracking the loaded hormone drug *in vivo*. The mesoporous silica layer has a high loading efficiency for E_2_ and provides a relatively long-lasting drug release within 50 h. EDTA anchored on the silica layer endows the nanocomposite with a bone targeting property. The nanocomposite effectively reverses estrogen deficiency-induced osteoporosis and reduces the damage of hormone to the uterus. The bone mineral density in the nanocomposite treatment group is nearly twice that of the ovariectomized (OVX) group. Compared with the E_2_ group, the uterine weight and luminal epithelial height were significantly lower in the nanocomposite treatment group.

**Conclusion**: This work demonstrated that E_2_-csUCNP@MSN-EDTA alleviates the side effect of hormone therapy while maintaining its therapeutic efficacy, which has great potential for developing the next generation of methods for osteoporosis treatment.

## Introduction

Osteoporosis is a skeletal disease associated with a decline in bone mineral density (BMD) and has a negative impact on bone microarchitecture. Its prevalence is as high as 50% in postmenopausal women 1. Drugs are currently the first treatment choice with great effect on the treatment of osteoporosis; however, the target organs are diverse, and thus the adverse reactions caused by poor targeting are increased. Specifically, one of the most representative drugs is 17β-estradiol (E_2_) 2. It has been reported that E_2_ could reduce the occurrence of osteoporotic fractures by approximately 50% in menopausal women 3 However, the conventional administration mode of E_2_ results in nonselective accumulation in organs such as the breast, heart and uterus, and this accumulation may cause severe side effects such as cardiovascular disease and high cancer risk in the breast, ovaries, and endometria 4-6. Reducing the non-specific enrichment of hormone drugs in non-target tissues and improving their bone-targeting ability is extremely important for broadening the clinical applications of E_2_. Recently, nanomaterials emerged as ideal drug carriers for reducing the exposure of hormone drugs to non-target tissues and improving their therapeutic efficacy 7, 8. For example, Lu *et al.* reported an ethinylestradiol-encapsulating liposome nanoparticle that has better osteoblast activation effect than free ethinylestradiol 9, but the uptake of the liposomes by bone has not been fully investigated.

Upconversion nanoparticles have great potential for diagnostic and therapeutic applications for some diseases such as neurodegenerative diseases and some cancer metastasis 10-13. Upconversion nanoparticles are nanoscale crystals doped with rare earth ions and have a unique optical property of converting low energy excitation into high energy emission 14. As promising bioimaging agents, their advantages include optical stability, low background noise, non-photobleaching, high luminescence signals, minimized photodamage to biological tissue 15, 16. In addition, their easy-to-modify surface makes them promising nanocarriers for loading and delivering drugs and improving therapy efficiency 11, 17, 18. The surface modifications promote the processes of cellular interaction, endocytosis, tissue targeting, intracellular transport and so on 18-20. Among the various surface modifications strategies, mesoporous silica coating approach is a classical method for preparing drug delivery systems, which exhibits profound application prospects because of the unique advantages of mesoporous silica nanomaterials (MSNs), such as large specific surface area, adjustable pore size and excellent biocompatibility 21-24. Nano-drug carriers based on MSNs have been widely studied in the field of biological medicine, especially as an antitumor-drug carrier 10, 21, 25. In addition, they have been considered a new approach to improve the oral bioavailability of certain insoluble drugs (carvedilol and fenofibrate) in the treatment of certain cardiovascular or inflammatory diseases 26. Well-tailored MSNs can also control the release of ibuprofen, which is one of the commonly accepted non-steroidal antipyretic analgesics 27.

Several studies have reported that upconversion nanoparticles showed great distribution in bone *in vitro*, and we intend to further improve its bone-targeting property and investigate *in vivo* efficacy and safety 9, 12, 14. Herein, we are interested in developing a drug delivery nanosystem to achieve high bioavailability of E_2_ for improving therapeutic efficacy and minimizing negative effects while visualizing the real-time biodistribution of E_2_.

## Results and Discussion

### Synthesis and characterization of upconversion nanocomposite

**Scheme 1:**
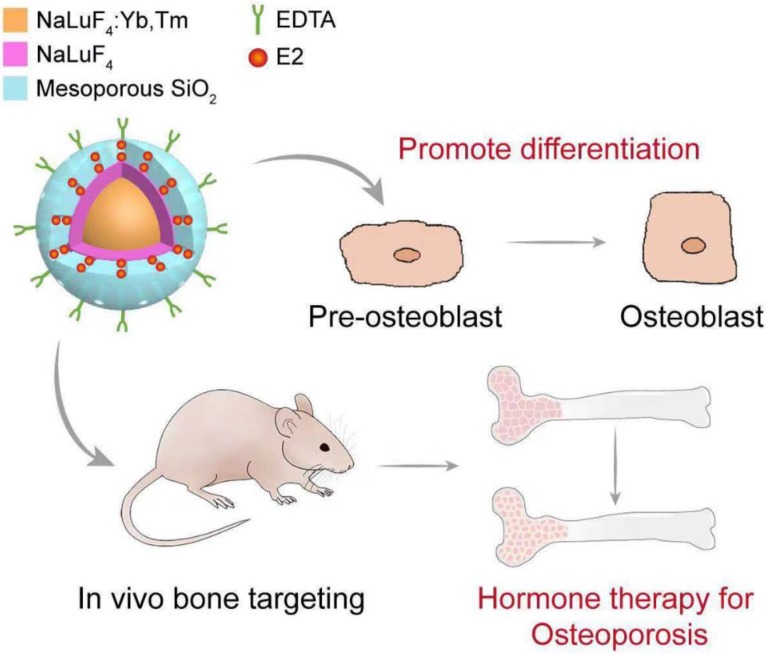
Schematic illustration of the structure of upconversion nanocomposite and its application in bone-targeted hormone replacement therapy for osteoporosis.

**Figure 1:**
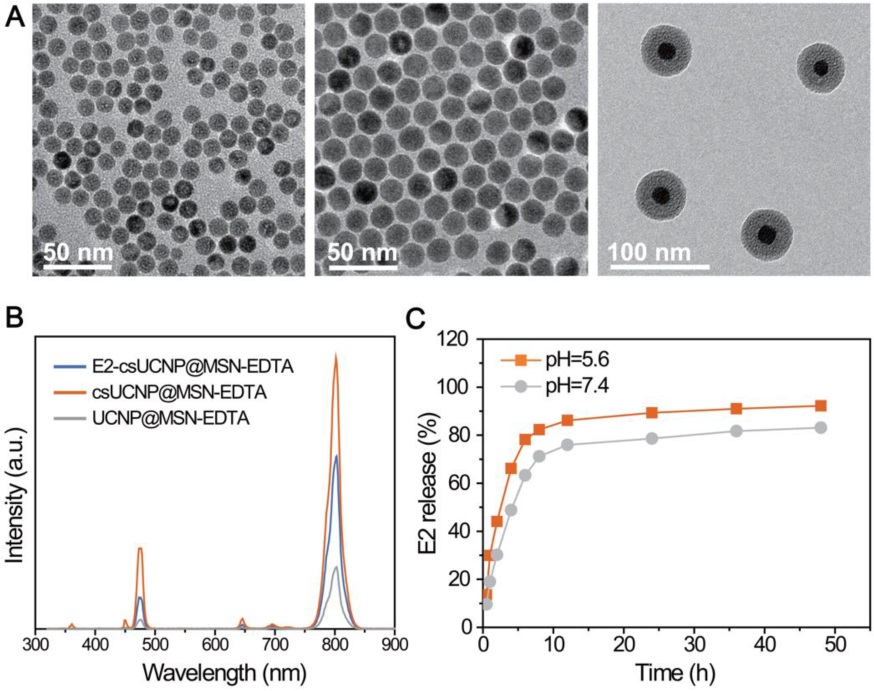
Characterizations of UCHRT. (A) TEM images of NaLuF_4_:Yb,Tm nanoparticles, NaLuF_4_:Yb,Er,Tm@NaLuF_4_ nanoparticles and NaLuF_4_:Yb,Tm@NaLuF_4_@mSiO_2_. (B) Room-temperature UCL emission spectra of E_2_-csUCNP@MSN-EDTA, csUCNP@MSN-EDTA and UCNP@MSN-EDTA (doped with Tm^3+^) dispersed in water (2 mg/mL) under excitation at 980 nm (power density ≈ 800 mW). The E_2_ concentration of E_2_-UCNPs was calculated using the detailed titration spectra. (C) Release behavior of E_2_ from E_2_-csUCNP@MSN-EDTA in PBS solution.

We constructed an upconversion nanocomposite, NaLuF_4_:Yb,Tm@NaLuF_4_@mSiO_2_-EDTA-E_2_ (E_2_-csUCNP@MSN-EDTA, UCHRT), with an upconversion nanoparticle core and mesoporous silica shell layer. The nanostructure of this upconversion nanocomposite is shown in Scheme 1. Upconversion nanoparticles NaLuF_4_:20%Yb,1%Tm were synthesized by a well-established solvothermal method 28. As shown in Figure 1A, the nanoparticles with a diameter of ~14 nm were spherical in shape. To obtain core-shell NaLuF_4_:20%Yb,1%Tm@NaLuF_4_ nanoparticles (csUCNPs), NaLuF_4_ layer was grown on the surface of NaLuF_4_:20%Yb,1%Tm* via* the epitaxial growth method 29. After coating of the NaLuF_4_ layer, the size of the nanoparticles increased to ~20 nm. The mesoporous silica layer was then grown onto the surface of csUCNPs by using CTAB as the pore template 30, 31. The mesoporous silica layer was ~22 nm in thickness, and the porous structure was clearly seen in the TEM images. The X-ray powder diffraction (XRD) patterns of both NaLuF_4_:20%Yb,1%Tm and NaLuF_4_:20%Yb,1%Tm@NaLuF_4_ nanoparticles can be indexed hexagonal phase of NaLuF_4_. The diffraction peaks at 17.2°, 30.0°, 30.9° and 43.3° are attributed to the (1 0 0), (1 1 0), (1 0 1) and (2 0 1) lattice planes of hexagonal NaLuF_4_. Moreover, the mesoporous silica coating did not change the crystalline phase of the upconversion nanoparticles (Figure S1). Nitrogen absorption experiment was performed to characterize the pore size of csUCNP@MSN. As shown in Figure S2, there are two pore distribution peaks centered at 1.4 nm and 3.5 nm in csUCNP@MSN sample, which indicates the existence of mesoporous structure of the nanocomposite. Ethylenediaminetetraacetic acid (EDTA) was conjugated on the silica layer which was characterized by Fourier-transform infrared spectroscopy **(Figure S3)**. A strong and wide band of O-H stretch centered at 3413 cm^-1^ and C=O stretch centered at 1654 cm^-1^ can be observed in csUCNP@MSN-EDTA sample (Figure S3B), indicating the existence of carboxyl group from EDTA.

To demonstrate that UCHRT nanocomposite can be used for *in vivo* bioimaging, the upconversion luminescence (UCL) of UCHRT was investigated. The luminescence spectra of csUCNP@MSN-EDTA exhibit the characteristic emission (^1^G_4_→^3^H_6_) in blue spectral region and (^3^H_4_→^3^H_6_) in near infrared range of Tm^3+^-doped upconversion material. After E_2_ loading, the upconversion emission of UCHRT was reduced to ~70% of that of csUCNP@MSN-EDTA (Figure 1B), but the emission was still strong enough for further bioimaging. It is worth noting that the interlayer of NaLuF_4_ plays an important role in protecting the upconversion luminescence. As shown in Figure 1B, NaLuF_4_:Yb,Tm core nanoparticles (UCNP) without the NaLuF_4_ layer protection were directly coated with mesoporous silica (NaLuF_4_:Yb,Tm@meso-SiO_2_-EDTA represents UCNP@MSN-EDTA), the luminescence had a 4.4-fold reduction when comparing it to its counterpart with the NaLuF_4_ protection layer (csUCNP@MSN-EDTA).

The loading efficiency of E_2_ was determined by UV-Vis absorption spectroscopy. As shown in Figure S4, the final loading efficiency of E_2_ in the csUCNP@MSN-EDTA is 14.5 wt%. We then studied the release behavior of E_2_ from csUCNP@MSN-EDTA nanocomposites. E_2_-csUCNP@MSN-EDTA was dispersed into two types of buffer solutions at pH 5.6 and 7.4. The release of E_2_ was also determined by UV-Vis absorption spectra. As shown in Figure 1C, the release of E_2_ is slightly faster in the acidic environment (pH = 5.6) than in neutral condition (pH = 7.4). This is useful for achieving a higher release of E_2_ in the cell, as acidic endosome or lysosome will trigger the release of E_2_ when E_2_-csUCNP@MSN-EDTA nanocomposites are internalized by cells.

### Osteogenic behavior *in vitro*

#### Tracking of upconversion nanoparticles in MC3T3-E1 cells

**Figure 2:**
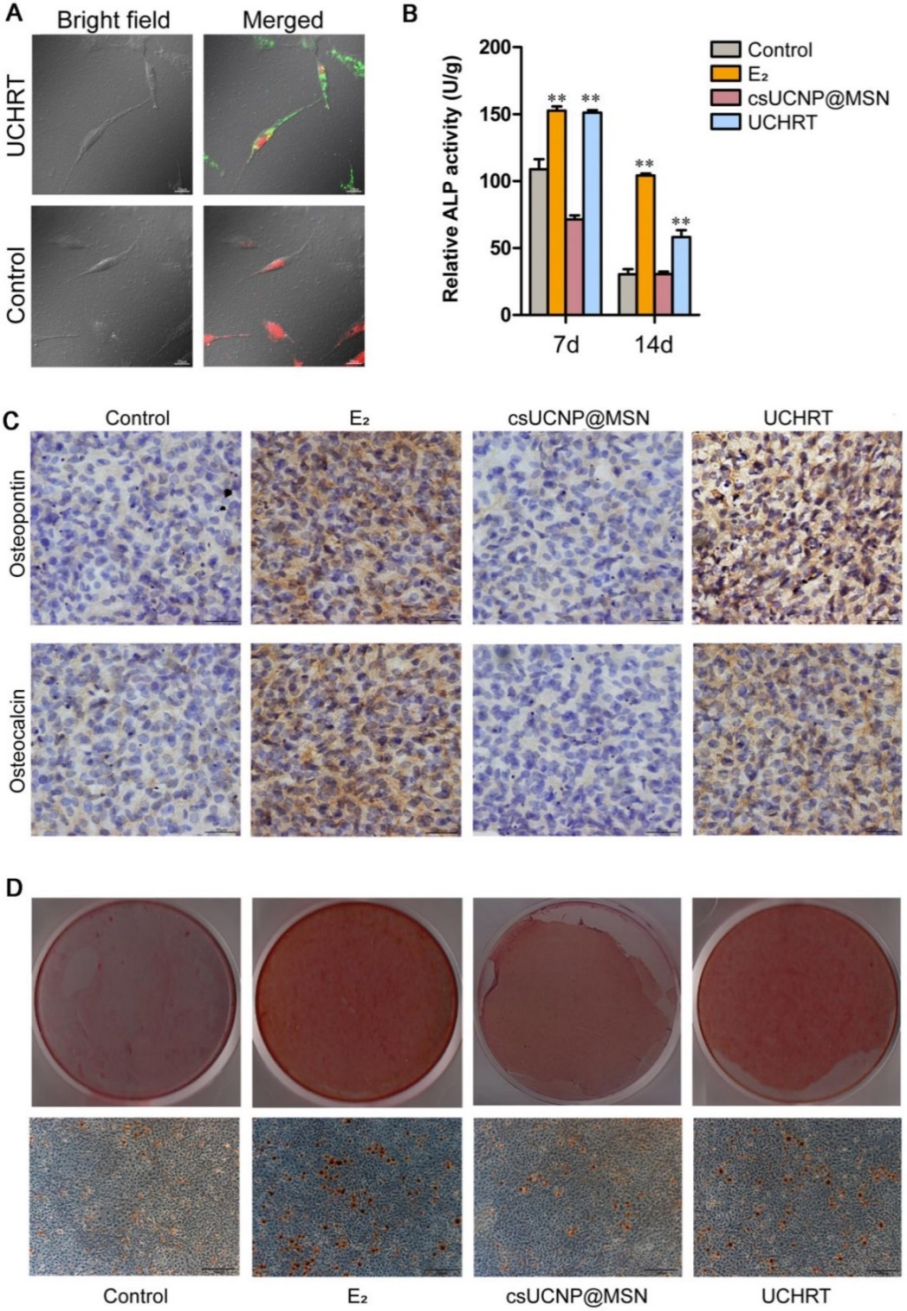
Effects of UCHRT *in vitro*. (A) LSUCLM images of fixed MC3T3-E1 cells incubated with UCHRT (200 μg/mL) or PBS (Control group) for 3 h with CW 980 nm (power density ≈ 800 mW) laser. (B) MC3T3-E1 cells were treated with E_2_, UCHRT and csUCNP@MSN respectively. After 7 and 14 days, ALP activity was analyzed. The ability of forming mineralized nodules can reflect the late osteogenic differentiation ability. (C) Immunohistochemical staining of MC3T3-E1 cells after 14 days of treatment. The deeper the staining, the greater the amount of expression. (D) Bright-field and microscopic images of MC3T3-E1 cells after 21 days of treatment. **, *P* < 0.01, compared with the control group.

The cellular uptake capability of nanoparticles is significant for their ability to accurately deliver and release E_2_ to the skeleton. Therefore, an affinity assay between UCHRT and preosteoblast cells or skeleton tissue was performed. At the cell level, UCL signals (highlighted in green) were observed (Figure 2A and S5) in propidium iodide (PI) stained cells after incubation for 3 hours. The content of nanoparticles in the cell lysate was measured. The cell uptake ratio of UCHRT to csUCNP@MSN treatment was 4.3, which revealed that UCHRT has higher affinity to preosteoblast cells than csUCNP@MSN. These results demonstrated that UCHRT nanoparticles were successfully taken up into MC3T3-E1 preosteoblast cells.

#### Osteogenesis of MC3T3-E1 preosteoblasts

To examine the osteogenic differentiation ability of UCHRT *in vitro*, we first measured the expression levels of osteogenic markers, including alkaline phosphatase (ALP), osteocalcin (OCN), and osteopontin (OPN). These osteogenic markers can reflect the osteogenic ability, that is, the higher the expression of the marker means the stronger osteogenic differentiation ability. ALP is one of the excellent indices in the early stage of osteogenic differentiation. We found that E2 group (positive control) and UCHRT group induced significant increase of ALP activity, while csUCNP@MSN and control group did not induce any changes both at 7 and 14 days **(Figure 2B)**. Similar results were obtained after staining of ALP at 7 days **(Figure S6)**. Likewise, the expression levels of OPN and OCN were higher in cells incubated with E_2_ and UCHRT compared with those of the control groups **(Figure 2C)**. Next, we analyzed the late stage osteogenic differentiation of MC3T3-E1 preosteoblast cells by Alizarin red staining. Calcium deposition (mineralization) is a specific indicator of advanced osteogenesis formation, so the ability to form mineralized nodules can reflect the late osteogenic differentiation ability. As shown in **Figure 2D**, UCHRT nanoparticles induced an increase of mineralization compared with the control group and csUCNP@MSN group.

### *In vivo* behavior of preventing osteoporosis

#### *In Situ* and *Ex Vivo* UCL Imaging

**Figure 3:**
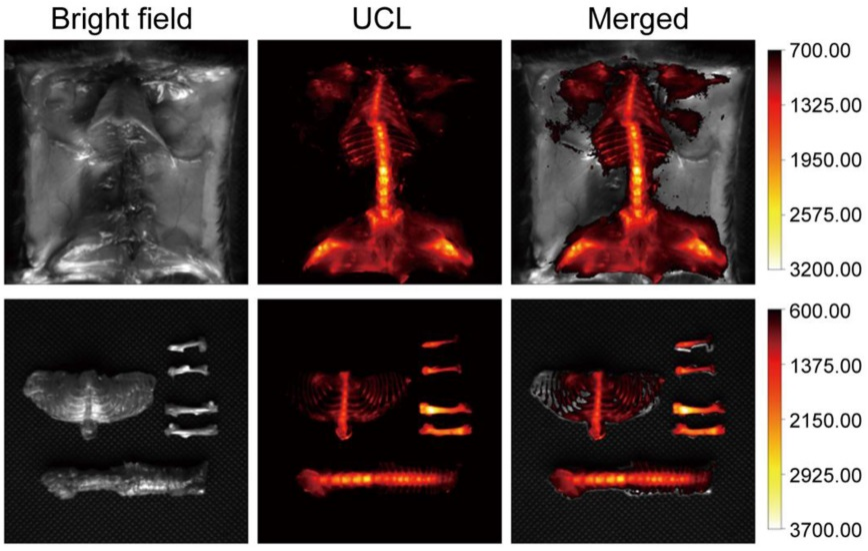
*In situ* and *ex vivo* imaging. Kunming mice were intravenously injected with UCHRT for 6 h. *In situ* and *ex vivo* UCL images of bone tissue were shown.

To demonstrate that our composite can be used for *in vivo* bioimaging, we conducted a whole body UCL imaging experiment after 6 hours post-injection. Compared with PBS and csUCNP@MSN injected groups, UCL signals were distributed over the whole body (Figure 3, Figure S7, Figure S8 and Figure S9). Another experiment was carried out to further confirm the bone affinity of UCHRT. Bones extracted from mouse were immersed in UCHRT and csUCNP@MSN dispersion respectively. After rinsing the bones with deionized water, UCHRT immersed bones exhibited obvious upconversion luminescence signals while the counterparts immersed in csUCNP@MSN had almost no signals (**Figure S8**). The ratio of UCHRT to csUCNP@MSN treatment was 5.5. These results demonstrated that the UCHRT have the ability to target bone, especially the bilateral femur and lumbar vertebra.

#### Protective effect on osteoporosis in ovariectomized mice

**Figure 4:**
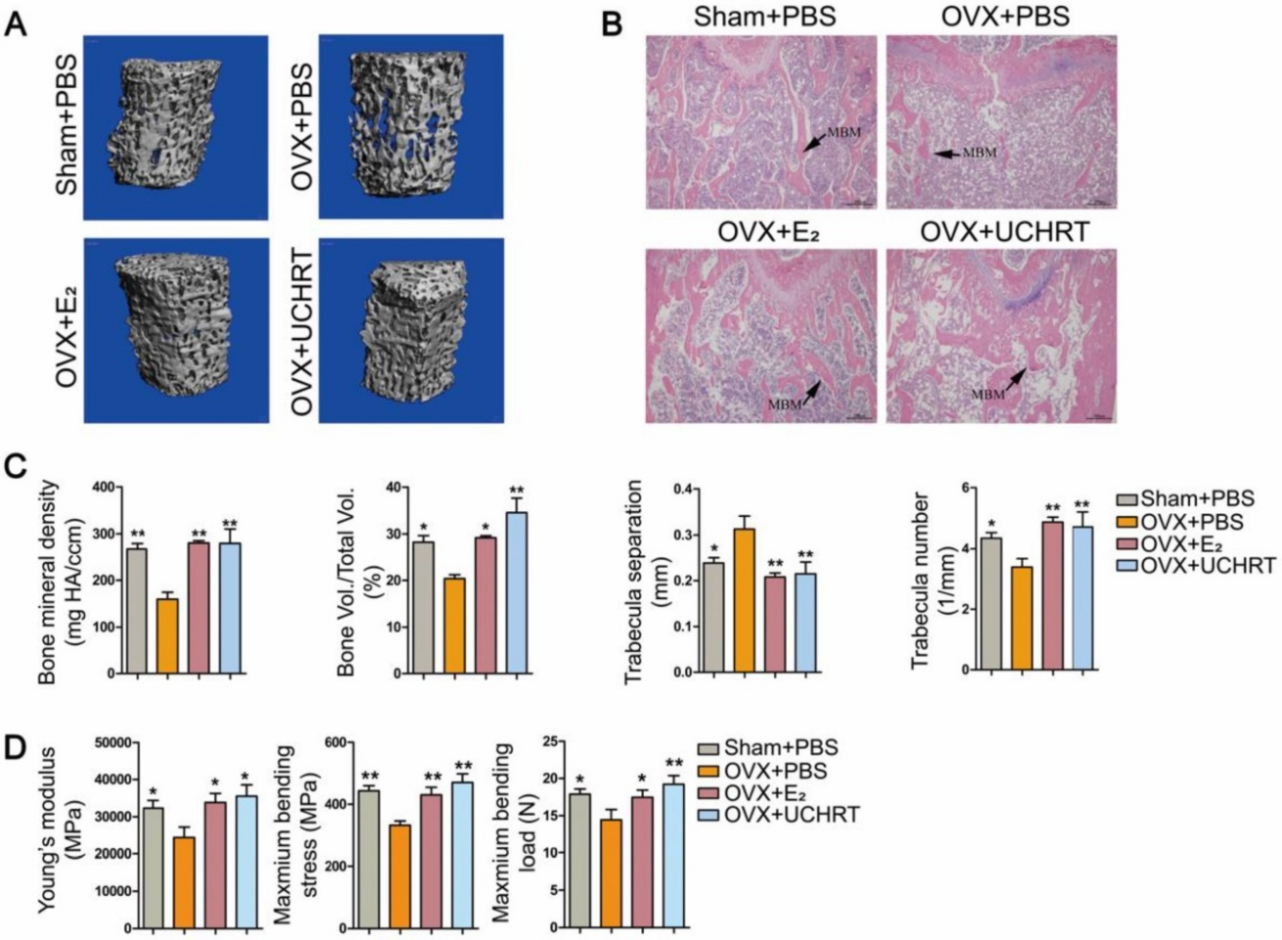
Effects of UCHRT *in vivo*. (A) Three-dimensional reconstruction of trabecular bone from the fourth lumbar vertebra of all the four groups. (B) H&E analysis -stained histological sections of femur. (C) Architectural parameters of BMD, BV/TV, Tb.N and Tb.Sp were shown. (D) Biomechanical test results of Young's modulus, maximum bending stress and maximum bending load. *, *P* < 0.05 and **, *P* < 0.01, compared with the OVX group.

**Figure 5:**
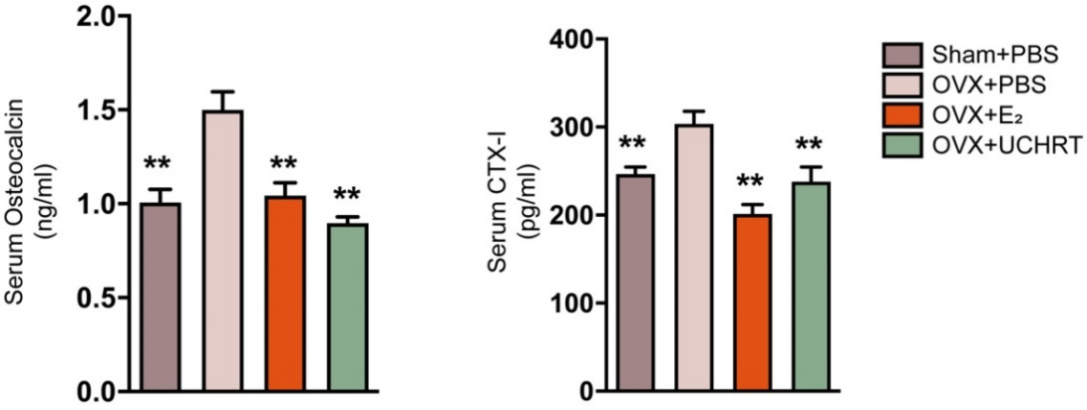
Changes of bone turnover biomarkers. Effects of UCHRT on bone turnover biomarkers in OVX mice, including serum OCN (A) and serum CTX-I (B) were shown. **, *P* < 0.01, compared with the OVX group.

We evaluated the preventing effect of UCHRT nanoparticles on osteoporosis induced by ovariectomy in mice. Ovariectomy can cause estrogen deficiency and then osteoporosis 32, 33. The typical changes of bone metabolic markers of osteoporosis including serum OCN and C-terminal telopeptide of collagen I (CTX-I), bone mineral density (BMD), bone morphometric parameters (BV/TV, Tb. N and Tb.Sp) and bone biomechanical properties can be observed in ovariectomized mice. Therefore, we tested the above indicators to determine the osteoporotic status. We first evaluated BMD and bone morphometric parameters using Micro-CT. Three-dimensional bone images and quantitative data on architectural parameters are shown in **Figures 4A and 4C**. We found that the OVX+PBS group showed a significant loss of bone mass compared with that of the other groups. The BV/TV, Tb. N values showed a significant reduction and Tb.Sp values showed a great increase in the OVX+PBS group, indicating osteoporosis. Compared to the OVX+PBS group, significantly higher values for BV/TV and Tb.N and lower values for Tb.Sp were observed in UCHRT treatment group. Additionally, the BMD (TV) of the lumbar vertebra in OVX+PBS mice was decreased compared with normal mice. However, the BMD significantly recovered approximately to normal in the E_2_ and UCHRT treatment group. The BMD, BV/TV, Tb.N and Tb.Sp in the UCHRT-treated group were similar to those in the E_2_-treated group. These results were also consistent with the H & E staining analysis which is used for the examination of histological changes **(Figure 4B)**. The analysis of the biomechanical properties of bone tissue under external force is an important means to evaluate the effectiveness of drugs in preventing osteoporosis. The results** (Figure 4D)** showed that Young's modulus, maximum bending stress and maximum bending load were significantly lower in OVX+PBS mice compared with those of the other groups. As is shown in **Figure 5**, serum OCN and CTX-Ι levels were significantly increased in the OVX+PBS group compared with those in the Sham group. In addition, the concentration of OCN and CTX-Ι were significantly decreased in both the E_2_ group and UCHRT group. Taken together, the osteoporotic markers of serum OCN and CTX-Ι showed a recovery in the OVX+PBS mice after treatment with E_2_ and UCHRT. These data indicated that UCHRT treatment improves the strength properties as well as E_2_.

#### Cytotoxic effect and side effect on uterus

**Figure 6:**
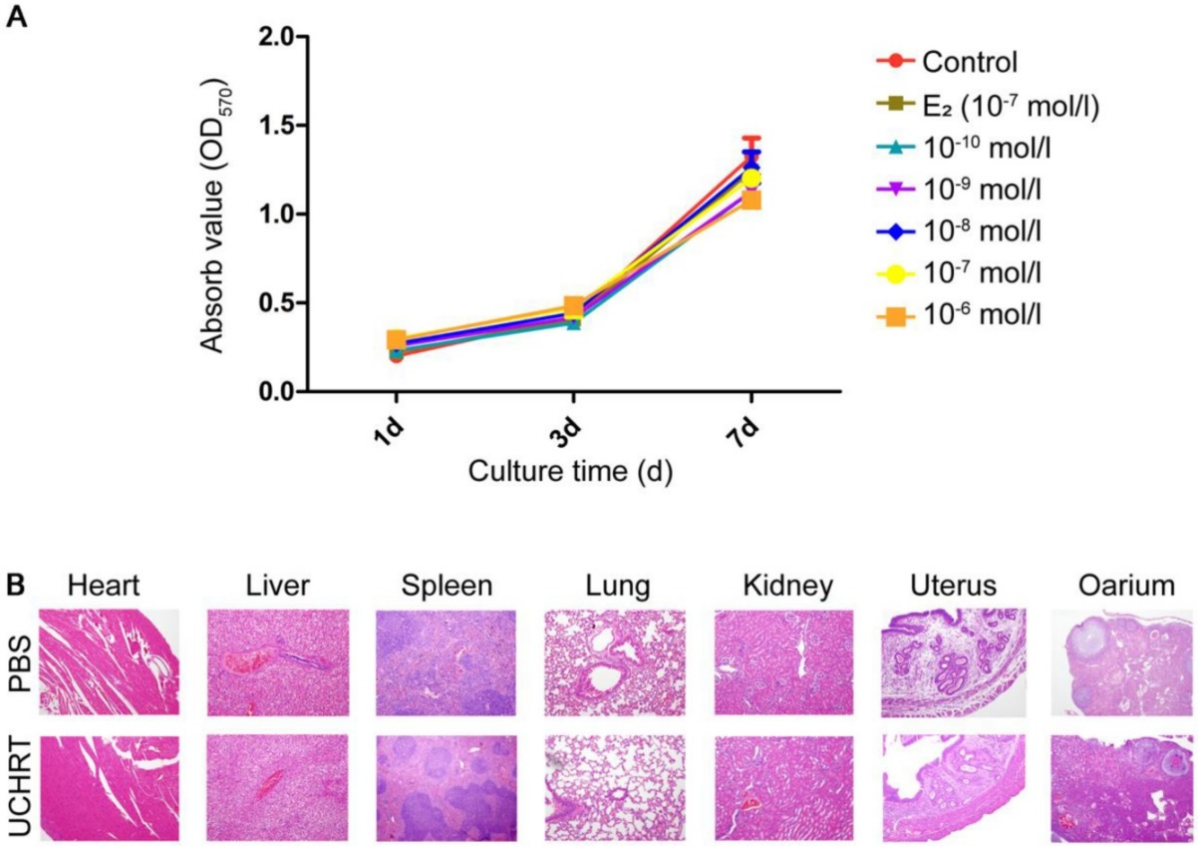
Toxicity of UCHRT. (A) Cell viability of MC3T3-E1 cells after nanoparticles treatment. Cells were treated with different inner E_2_ concentrations for 1, 3 and 7 days. (B) H&E analysis of major tissues after 24 h of injection of PBS or UCHRT.

**Figure 7:**
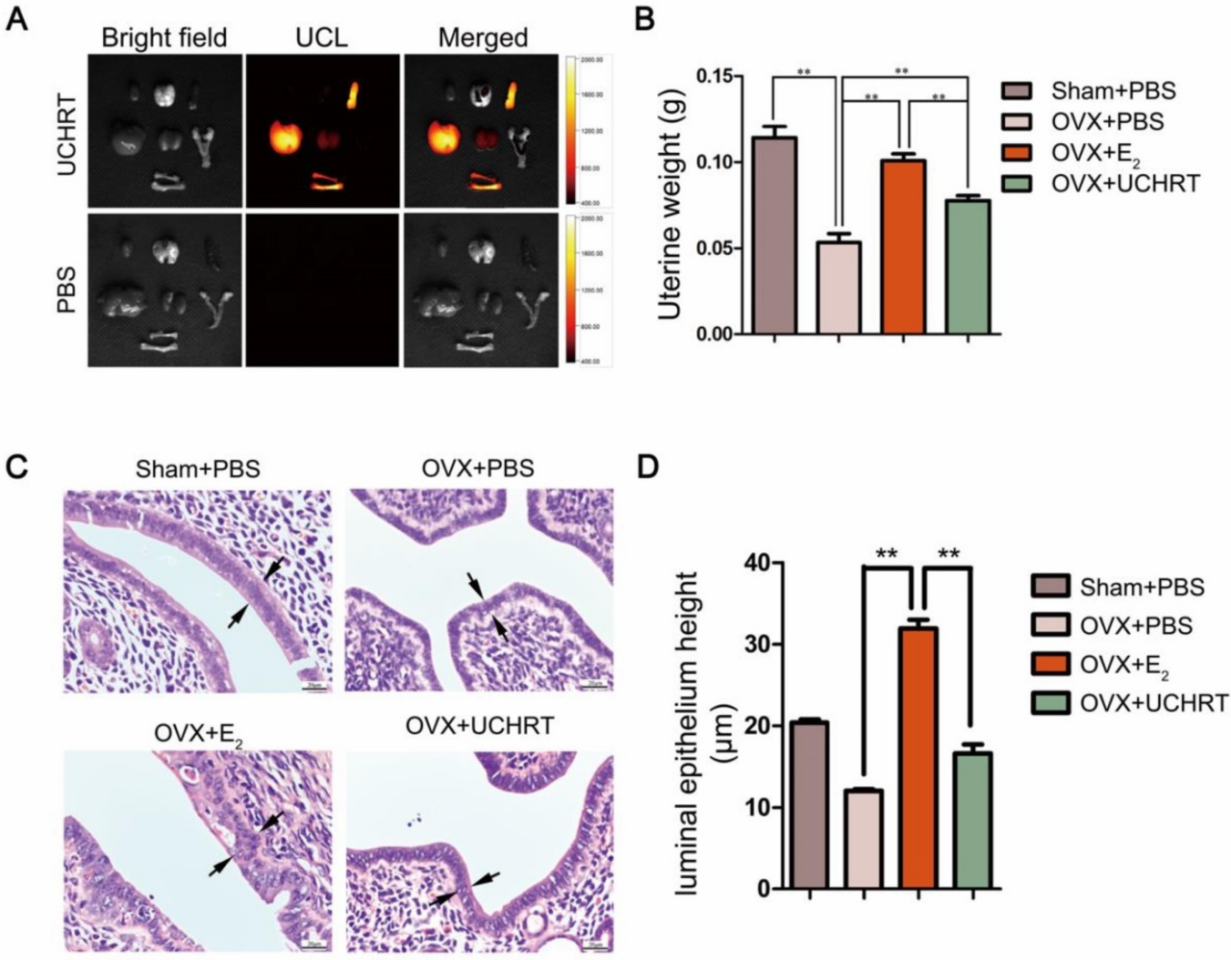
Effects of UCHRT treatment on the uterus in OVX mice. *In situ* and *ex vivo* UCL images of the main organs 6 h after intravenous injection. (A) UCHRT, 200 μg/m and PBS. (B) The wet weight of the atrophic uterus decreased, while the high dose of estrogen for a long time resulted in endometrial hyperplasia and increased wet weight. After the 8-week treatment, uterine weight was significantly lower in OVX mice and UCHRT exerted less influence on uterine weight than E_2_. *, *P* < 0.05 and **, *P* <0.01. (C) Representative images of effect of UCHRT on uterus histomorphometric after H&E staining. Luminal epithelium was shown as the black arrow. (D) Luminal epithelium height in each group (**, *P* < 0.01, compared with the E_2_ group).

Drug targeting refers to the delivery of therapeutic agents to osteoporotic bone or other target tissues, as well as the reduction of nonspecific interactions and systemic toxicity through specific drug delivery systems. No drug can exert positive effects if it is off target. EDTA, a small molecule with a calcium affinity and good biological safety, was conjugated on the silica layer to control the nucleation. In addition, it can be used for targeting hydroxyapatite (HAp), which is the main inorganic composition of animal and human bones 34, 35. In recent years, some researchers have used EDTA-modified nanopharmaceuticals for calcium-deposited aneurysms targeting to promote the treatment effect 36. Ito* et al.* 37 showed that HAp scaffold material modified by EDTA could significantly promote the osteogenic action of osteoblasts MG63. Here, we use EDTA to modify the E_2_-loaded nanoparticles and we speculated that EDTA modification could enhance the bone targeting ability of nanoparticles, thus reducing uterine side effects. First, our results showed there was no cytotoxicity to MC3T3-E1 cells *in vitro* and to the main organs *in vivo* was observed after treatment **(Figure 6)**. Then, the tissue distribution of UCHRT within the dissected organs was analyzed, and UCL images and ICP-AES data of the main organs showed the accumulation of nanoparticles in the uterus was low **(Figure 7A, Figure S10 and Figure S11)**. More importantly, OVX can cause significant atrophy of the uterus. On the contrary, too much E_2_ treatment can lead to endometrial hyperplasia. **Figure 7B** shows that the uterine weight was significantly decreased in OVX+PBS mice compared to the other three groups, and there was a significant difference between the groups treated with E_2_ and UCHRT. H & E staining of the uterus showed luminal area and luminal epithelial cells. E_2_ treatment showed significant increase in luminal epithelium height compared with Sham group and OVX+PBS group. A reduction in the luminal epithelium height was observed in the OVX+PBS group and UCHRT group **(Figure 7C, 7D and Figure S12).** These results indicated that UCHRT can target bone tissue and have less interaction with uterine tissue, and thus reducing the side effects of E_2_.

To date, some carrier molecules have been used to formulate drug delivery or release for E_2_, including, nanomaterials, tissue selective estrogen complexes (TSEC), estrogen analogue targeting vector (aspartic acid (Asp), arginine-glycine-aspartic acid (RGD) peptides, tetracyclines and so on) 14, 21, 38-40. Asp, RGD peptides and tetracyclines are carriers with bone tissue affinity 41. After binding with E_2_, they not only selectively target bone tissue, but also prolong drug action time and effectively prevent bone loss 39, 42. However, the disadvantages of these molecules are that they cannot achieve controlled release, and they cannot maintain the stable drug bioactivity 42. However, a nano drug delivery system can solve both problems at the same time. In our study, UCHRT can achieve a high loading rate and can control E_2_ release to maintain its biological activity for long period of time in the complex internal environment. In our previous work, we developed 17β-estradiol-loaded PEGylated upconversion nanoparticles (E_2_-UCNP@pPEG) and our results showed that E_2_-UCNP@pPEG provides an effective system for E_2_ delivery with a slow controlled release 17. On the basis of previous work, we further anchored the nanoparticles with EDTA, which is well known for its ability of calcium affinity and bone-targeting. Therefore, the nanosystem can target bone and achieve efficient theranostic effects for osteoporosis. Theranostic effects of the nanoparticles were not investigated in the previous work, and here, we determined UCHRT was able to prevent estrogen deficiency induced osteoporosis *in vivo*, extending the medical application of nanoparticles. Additionally, Hu *et al.* prepared mesoporous silica nanoparticles to enhance the loading efficiency of E_2_. The authors found that their nanoparticles effectively stimulate the mineralization capability of osteoblasts 43. Wang *et al*. 14 reported that such strategy can be used to prepare a multifunctional mesoporous bioactive glass (MBG)/UCNP nanocomposite with the ability to deliver anti-cancer drugs, monitor drug release and promote osteogenic differentiation. However, *in vivo* therapy was not demonstrated in that work due to the lack of bone targeting moieties on the nanoparticles. In our study, the biological effects *in vitro* and *in vivo* were both investigated, and we found that UCHRT shared similar effects on osteogenic differentiation and osteoporosis prevention with E_2_ while having fewer side effects on uterus.

## Conclusions

In summary, we successfully fabricated an UCHRT nanocomposite that serves as a drug-delivery system and analyzed its effect to promote osteogenic differentiation *in vitro* and prevent osteoporosis *in vivo*. The nanoparticles exhibited great ability for drug loading, and the ability to sustain E_2_ release E_2_ as well as promote osteogenic differentiation and reduce bone loss, while exerted less side effects on uterus than E_2_. Based on these characteristics, the UCHRT nanoparticles we presented here will hopefully be used as an alternative therapeutic agent to maintain bone homeostasis. This work points to a promising strategy for preventing postmenopausal osteoporosis with upconversion nanocomposites and will provide inspiration for hormone drug delivery in the future.

## Materials and Methods

### Characterization

The size and morphology of nanoparticles were characterized by FEI Tecnai G2 20 TWIN transmission electron microscope (TEM) at 200 kV. TEM samples were prepared by dipping the copper grid into a diluted nanoparticles' solution. Powder X-ray diffraction (XRD) measurements of the nanoparticle samples were conducted on a Bruker D4 diffractometer (Cu Kα radiation, λ = 1.54056 Å) of which the 2θ range is from 10 to 90° and the scanning rate is 0.5°/min. Fourier transform infrared (FTIR) spectra were collected using an IRPRESTIGE-21 spectrometer (Shimadzu). Nanoparticles were mixed with KBr and tableted into pellet to make FTIR samples. E_2_ release studies were carried out on Shimadzu UV 2550 spectrometer. The upconversion luminescence (UCL) spectra were collected by using Edinburgh FLS-920 spectrometer equipped with an excitation source of 0-3 W adjustable 980 nm semiconductor laser (Connet Fiber Optics, China). All the photoluminescence studies were carried out at room temperature.

### Synthesis of UCHRT

#### Synthesis of NaLuF_4_:Yb,Tm nanoparticles (UCNP)

NaLuF_4_:Yb,Tm upconversion nanoparticles were prepared by a solvothermal method. In a typical synthetic process, 1 mmol of lanthanide chloride (LnCl_3_), of which the ratio of LuCl_3_, YbCl_3_ and TmCl_3_ is 0.79:0.2:0.01, was loaded into a three-necked flask. After that, 6 mL of oleic acid and 15 mL of 1-octadecene were mixed with LnCl_3_. The mixture was blended by magnetic stirrer and heated to 160°C to obtain a transparent solution. Then, the solution was cooled down to 90°C by turning off the heating device. Sodium and fluoride source for upconversion nanoparticles were prepared by dissolving 2.5 mmol NaOH and 4 mmol NH_4_F in 5 mL methanol. The methanol solution was added dropwise into the solution containing LnCl_3_. The mixture was degassed for 30 min at 90°C and nitrogen was used as protective atmosphere for the system. Afterward, the solution was heated to 300°C in 20 min and the temperature was maintained for 1 h. When the reaction was finished, the solution was cooled down to room temperature. Nanoparticles were precipitated by pouring 10 mL ethanol into the reaction solution and were collected by centrifugation. To remove excessive organic compounds in the nanoparticles, the nanoparticles were washed by ethanol/cyclohexane (1:1 v/v) for three times. Finally, nanoparticles were dispersed in 5 mL of cyclohexane for the following synthesis.

#### Synthesis of NaLuF_4_:Yb,Tm@NaLuF_4_ nanoparticles (csUCNP)

NaLuF_4_ shell layer was coated on NaLuF_4_:Yb,Tm nanoparticles by an epitaxial growth method. The synthetic procedures are similar to the synthesis of NaLuF_4_:Yb,Tm nanoparticles. Typically, 1 mmol LuCl_3_ was mixed with 6 mL oleic acid and 15 mL 1-octadecene in a three-necked flask. The system was heated to 160°C under continuous stirring. After 30 min, the system turned from turbid to transparent. NaLuF_4_:Yb,Tm nanoparticles previously dispersed in 5mL cyclohexane was added dropwise into the system. The system was kept at 80°C for 30 min to evaporate the cyclohexane. After that, methanol solution containing 2.5 mmol NaOH and 4 mmol NH_4_F was slowly added into the system under vigorous stirring. The reaction solution was degassed at 90°C for 30 min, and finally heated to 300°C for 1 h with nitrogen as protective atmosphere. The as-prepared NaLuF_4_:Yb,Tm@NaLuF_4_ nanoparticles (csUCNP) were precipitated by pouring 10 mL ethanol into the reaction solution and were collected by centrifugation. Ethanol/cyclohexane (1:1, v/v) was used to wash the nanoparticles. The purified nanoparticles were dispersed in cyclohexane for mesoporous silica coating.

#### Synthesis of NaLuF_4_:Yb,Tm@NaLuF_4_@Meso-SiO_2_-NH_2_ nanoparticles (csUCNP@MSN-NH_2_)

Ten milligrams of csUCNP dispersed in 1 mL cyclohexane was added dropwise into 10 mL aqueous solution containing 0.1 g CTAB. The mixture was stirred vigorously at room temperature overnight to evaporate the cyclohexane. The solution became transparent the second day indicating that cyclohexane was removed completely. After that, 20 mL deionized water were mixed with the solution followed by the addition of 3 mL ethanol and 0.12 mL NaOH solution (2 mol/L). Upon stirring, 160 μL of tetraethyl orthosilicate (TEOS) and 20 μL of (3-aminopropyl)triethoxysilane were injected into the reaction solution and the temperature of the mixture was elevated to 70°C. The temperature was maintained for 2 h to achieve mesoporous silica coating. The as-prepared csUCNP@MSN-NH_2_ nanoparticles were collected by centrifugation and washed with ethanol for 5 times. Then, the nanoparticles were dispersed into 50 ml ethanol solution containing 0.3 g NH_4_NO_3_ and heated to 60°C for 2 h to remove CTAB in the mesoporous structure.

#### Synthesis of NaLuF_4_:Yb,Tm@NaLuF_4_@Meso-SiO_2_-EDTA nanoparticles (csUCNP@MSN-EDTA)

Ten milligrams of csUCNP@MSN-NH_2_ nanoparticles were added into 10 mL of ethanol/acetic acid solution (1:1 v/v). Then, 30 mg ethylenediaminetetraacetic dianhydride was added into the solution. The solution was heated to 80°C for 2 h. The obtained csUCNP@MSN-EDTA nanoparticles were washed with ethanol for 5 times and re-dispersed in ethanol for further E_2_ loading.

### E_2_ loading on NaLuF_4_:Yb,Tm@NaLuF_4_@Meso-SiO_2_-EDTA nanoparticles (UCHRT)

Firstly, 2.8 mg E_2_ was dissolved into 5 ml ethanol, and then 10 mg csUCNP@MSN-EDTA nanoparticles dispersed in 5 ml ethanol was added into the solution and stirred at room temperature for 1 h. Then, the mixture was transferred to a rotary evaporator to evaporate the ethanol. The resultant nanoparticles were washed with acetone one time and DI water for two times. The final product was re-dispersed in saline to form a solution with a concentration of 2.5 mg/mL.

### Cell Culture

The pre-osteoblastic MC3T3-E1 cells were purchased from the cell resource center of Shanghai Institutes for Biological Sciences (SIBS). MC3T3-E1 cells were cultured in α-modified minimal essential medium (α-MEM; GIBCO) with 10% fetal bovine serum (FBS), 1% folic acid and inositol, 100 U/mL penicillin, and 100 μg/ml streptomycin. The cells were maintained at 37 °C in a humidified 5% CO_2_ atmosphere.

### Cytotoxicity

MC3T3-E1 cells were added with various dosages of UCHRT with inner E_2_ (Sigma-Aldrich, USA) at different concentrations (10^-10^, 10^-9^, 10^-8^, 10^-7^, and 10^-6^ mol/L). The concentration of E_2_ group was 10^-7^ mol/L. Cellular viability was then assessed with the 3-(4,5-dimethylthiazol-2-yl)-2,5 diphenyl tetrazolium bromide (MTT, Sigma-Aldrich, USA) kit, and the optical density at 450 nm was measured according to the manufacturer's instructions at days 1, 3 and 7. To further investigate its cytotoxicity *in vivo*, the major organs (heart, liver, spleen, lung, kidney, uterus and ovary) of mice were prepared for H&E histomorphology analysis 24 h after the intravenous injection of UCHRT (200 μL, 1.5 mg/mL) or PBS.

### UCHRT Uptake in MC3T3-E1 Cells

MC3T3-E1 cells were seeded in a confocal culture dish and treated with UCHRT. After incubating for 3 hours, the medium was discarded, and PBS was used to rinse the cells to remove residual nanoparticles. Propidium iodide (PI) was used to stain the nucleus. The uptake of UCHRT were observed with a modified Olympus FV1000 laser scanning upconversion luminescence microscope (LSUCLM) with a continuous-wave laser at 980 nm (Connet Fiber Optics, China) as the excitation source. UCL signals were collected with the excitation of 980 nm and collection of 500-560 and 600-700 nm after 3 hours of incubation. Cell nuclei stained by propidium iodide (PI) were observed under excitation of 515 nm and collection of 600-650 nm.

### UCL Imaging of UCHRT *In Situ* and* Ex Vivo*

*In situ* and *ex vivo* UCL imaging were carried out with a modified Kodak *in vivo* imaging system consisting of an external 0-5 W adjustable CW infrared laser (980 nm, Connet Fiber Optics, China) as the excited source and an Andor DU897 EMCCD as the signal collector. Kunming mice (6 weeks old), purchased from Shanghai Laboratory Animal Centre (Shanghai, China), were intravenously injected (IV) with UCHRT (200 μL, 1.5 mg/mL) or PBS. After 6 hours, the animals were euthanized. Bone and other major tissues were exposed for imaging.

ICP-AES is an important method for the determination of the microelement. Mice were intravenously injected (IV) with UCHRT (200 μL, 1.5 mg/mL) for 6 h. The animals were euthanized, and the main organs were used to indirectly analyze the distribution of the upconversion nanomaterials in the main organs by the ICP-AES method.

### Alkaline Phosphatase Activity and Staining Assay

MC3T3-E1 cells were seeded into 24-well plates at a density of 2×10^4^ cells per well. After 12 hours, cells were cultured with E_2_ (10^-7^ mol/L), UCHRT with an E_2_ concentration of 10^-7^ mol/L, or csUCNP@MSN. The ALP activity assay was performed on the 7th and 14th days of culture with an alkaline phosphatase activity kit (Beyotime Co., Haimen, China). The results were normalized to total cellular protein using a bicinchoninic acid (BCA) protein assay kit (Beyotime Co., Haimen, China) according to the manufacturer's protocol.

ALP staining was conducted with the BCIP/ NBT alkaline phosphatase staining assay kit (Beyotime Co., Haimen, China), in accordance with the manufacturer's protocol. MC3T3-E1 cells were seeded into 6-well plates at a density of 5 × 10^4^ cells per well. After treatment for 7 days, the cell was fixed with 4% paraformaldehyde for 15 min and washed with PBS. Then, cells were incubated with BCIP/NBT Alkaline Phosphatase Liquid Substrate in 37 °C for 30 minutes.

### Immunohistochemical staining

MC3T3-E1 cells were fixed with 4% paraformaldehyde and washed with PBS after 14 days of treatment. Then, cells were permeabilized with 1% NP-40 and blocked with 10% goat serum to reduce nonspecific staining, followed by incubation with anti-osteopontin (1:500 dilution; ab91655 for monoclonal antibody, Abcam) and anti-osteocalcin (1:150 dilution; sc30045 for polyclonal antibody, Santa Cruz Biotechnology) at 4ºC overnight. Then, cells were incubated with biotin-labeled secondary antibody (1:500 dilution; Santa Cruz Biotechnology, USA) for 1 h and then incubated with streptavidin-horseradish peroxidase (HRP) conjugate for 20 min. The presence of the expected protein was visualized by diaminobenzidine (DAB) staining and examined under a microscope (Epson Perfection 4990 Photo Scanner, USA).

### Alizarin Red Staining

The mineralization assay was performed on the 21st day of treatment. MC3T3-E1 cells were fixed with 4% paraformaldehyde for 15 min and rinsed twice with PBS (pH 4.2). Then, the fixed cells were stained with 2% Alizarin Red Solution (Sigma, USA) in 37 °C for 30 minutes followed by extensive washing by PBS. Images were obtained using a commercial digital camera and light microscopy analysis.

### Development of ovariectomized mice model and treatment

Forty 8-week-old female Kunming mice were purchased from Shanghai Laboratory Animal Centre (Shanghai, China). The mice were housed in an air-conditioned room at 23-25 °C with a 12 h light/dark cycle, and the mice had free access to food pellets and water. All the animal experiments were approved by the Ethics Committee of Xinhua Hospital Affiliated with the Shanghai Jiao Tong University School of Medicine. All the experimental procedures were performed in accordance with the Regulations for the Administration of Affairs Concerning Experimental Animals. The Kunming mice were anesthetized by intraperitoneal injection with a mixture of Rumpun (0.4 mL/kg) and Zoletil (0.6 mL/kg), and then OVX surgery was performed. The mice were divided into four groups and there are 10 mice in each group: Sham group (ovary intact) mice treated with PBS (Sham+PBS) and OVX mice treated with PBS (OVX+PBS), E_2_ (OVX+E_2_) and UCHRT (OVX+ UCHRT). The treatment was given as intraperitoneal injections every day for 8 weeks. The concentration of E_2_ in the E_2_ group and UCHRT group was 0.5 mg/25 g mouse.

### Markers of bone turnover

Serum C-terminal telopeptide of collagen I (CTX-I) and serum osteocalcin (OCN) were measured by enzyme-linked immunosorbent assay (ELISA) kits (Uscn Life Science, Wuhan, China) according to the manufacturer's protocol 2 months after OVX.

### Mechanical Testing

To evaluate the mechanical properties, the right tibias were tested under a three-point bending test machine (Instron Corporation, Norwood, MA, USA). Tibias were centered over two supports (10 mm apart) with a 1 N preload before loading to failure at a rate of 1 mm/min as the contact force. The outcome parameters that were calculated using the load deformation curve included the ultimate load (N), ultimate stress (MPa), and Young's modulus (MPa).

### Micro-CT analysis

The trabecular microarchitecture of the fourth lumbar (L4) vertebra of the mice was measured 2 months after OVX using a microcomputed tomography (Micro-CT) system (µCT 80; Scanco Medical, Zürich, Switzerland). The 3D images and the bone morphometric parameters of bone volume over total volume (BV/TV), trabecula number (Tb.N), trabecula separation (Tb.Sp) and BMD were determined by analyzing the volume of interest (VOI). The unit of measurement used was milligrams of hydroxyapatite (HA) per cubic centimeter (mg HA/ccm).

### H&E-Stained Tissues

The right femur and uterus were prepared for histomorphology analysis. The tissues were fixed in 4% paraformaldehyde at 4 °C for 48 h, and the femurs needed to be decalcified in 10% EDTA for 4 weeks. Next, we embedded the samples in paraffin for sectioning. HE staining was performed following the manufacturer's recommendations.

### Data Analysis

We used SPSS 20 (IBM Corp., USA) to analyze the collected data, which are presented as the means ± standard deviation (SD). Statistical evaluation was performed by a one-way analysis of variance among multiple groups. For all tests, a *P* value less than 0.05 was considered statistically significant.

## Supplementary Material

Supplementary figures.
