# Supplementary material for: EDTA-Modified 17β-Estradiol-Laden Upconversion Nanocomposite for Bone-Targeted Hormone Replacement Therapy for Osteoporosis
Source: Theranostics. 2020 Feb 10;10(7):3281–92. doi: 10.7150/thno.37599 (PMC7053193; doi:10.7150/thno.37599)
Supplement: Supplementary file 1 — Supplementary figures. [file thnov10p3281s1.pdf]

EDTA-Modified 17β-Estradiol-Laden Upconversion Nanocomposite for Bone-Targeted Hormone Replacement Therapy for Osteoporosis

Xiaoting Chen[a,†], Xingjun Zhu[b,†], Yan Hu[a], Wei Yuan[b], Xiaochen Qiu[b], Tianyuan Jiang[a], Chao Xia[a], Liqin Xiong[c], Fuyou Li[b,*], Yanhong Gao[a,*]

[a] Department of Geriatrics, Xinhua Hospital of Shanghai Jiaotong University, School of Medicine, Shanghai 200092, China.

[b] Department of Chemistry & Advanced Materials Laboratory, Fudan University, 220 Handan Road, Shanghai 200433, P.R. China.

[c] Shanghai Med-X Engineering Center for Medical Equipment and Technology, School of Biomedical Engineering, Shanghai JiaoTong University, Shanghai 200030, P. R. China

[†]These authors contributed equally.

[*] Corresponding author

Prof. Gao: gaoyanhong@xinhuamed.com.cn

Prof. Li: fyli@fudan.edu.cn

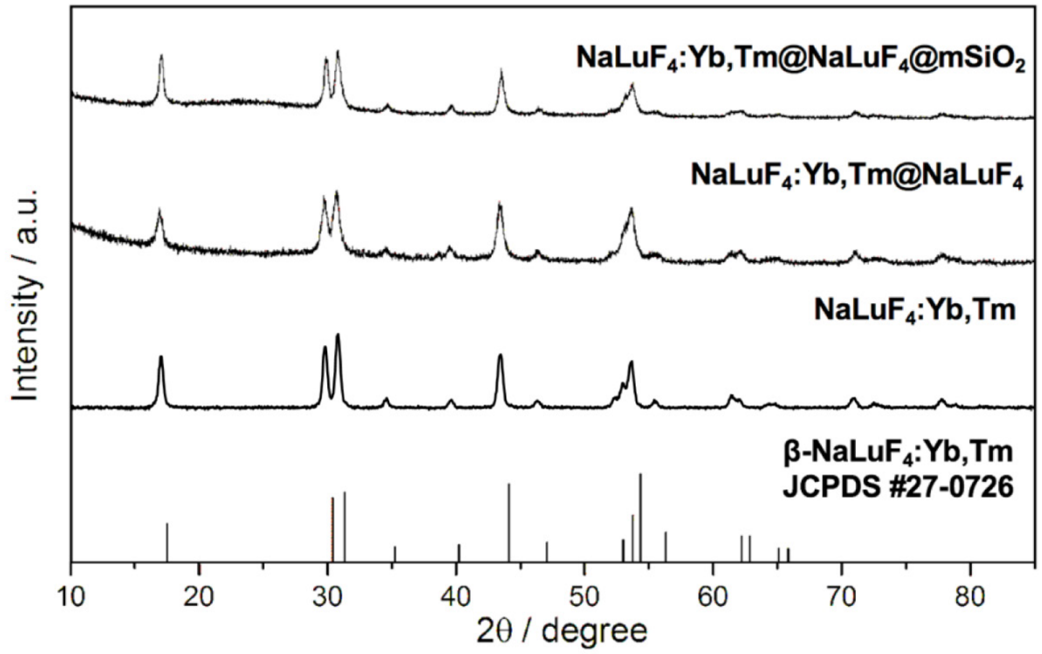

Figure S1. X-ray diffraction pattern of UCNPs $NaLuF_4$:20%Yb,1%Tm, $NaLuF_4$:Yb,Tm@$NaLuF_4$ and $NaLuF_4$:Yb,Tm@$NaLuF_4$@$mSiO_2$ nanoparticles. The standard patterns of β-$NaLuF_4$.

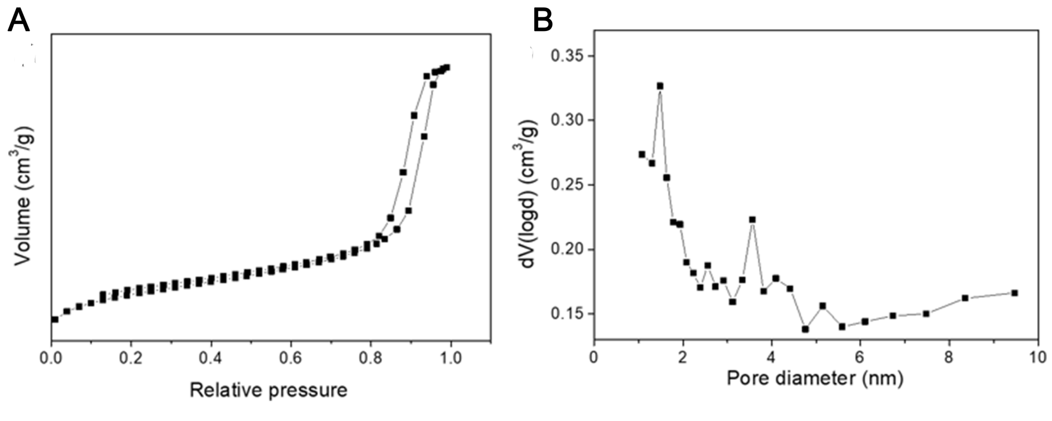

Figure S2. (A) Nitrogen adsorption–desorption isotherms and (B) Pore size distribution of csUCNP@MSN. The pore size is calculated by Barrett–Joyner–Halenda (BJH) model.

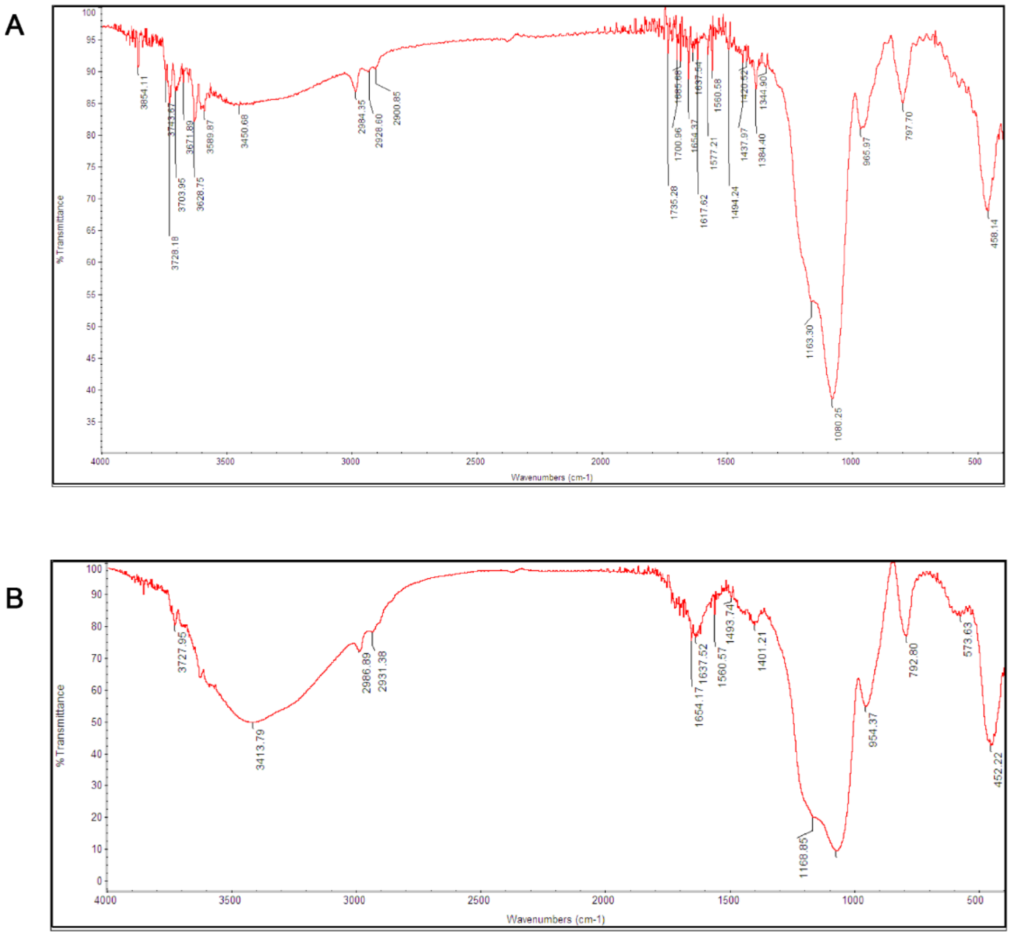

Figure S3. Room-temperature FT-IR spectra of csUCNP@MSN (A) and csUCNP@MSN-EDTA (B).

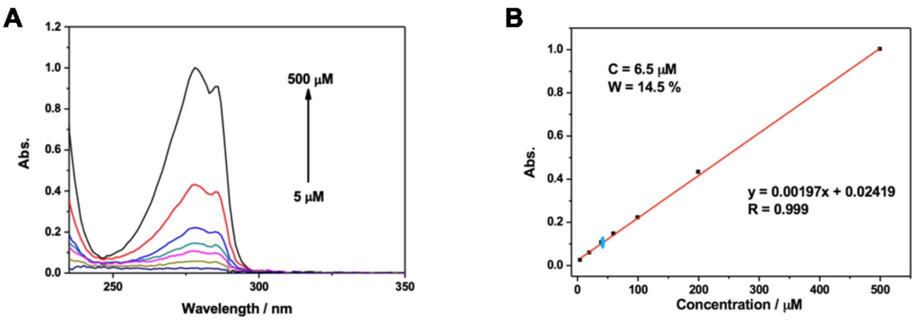

Figure S4. (A) UV/Vis absrption spectra of E_2 with different concentrations of 5-500 μM. (B) The absorbance at 278 nm as a function of E_2 concentration. The E_2 content on UCHRT was determined as 14.5 wt%.

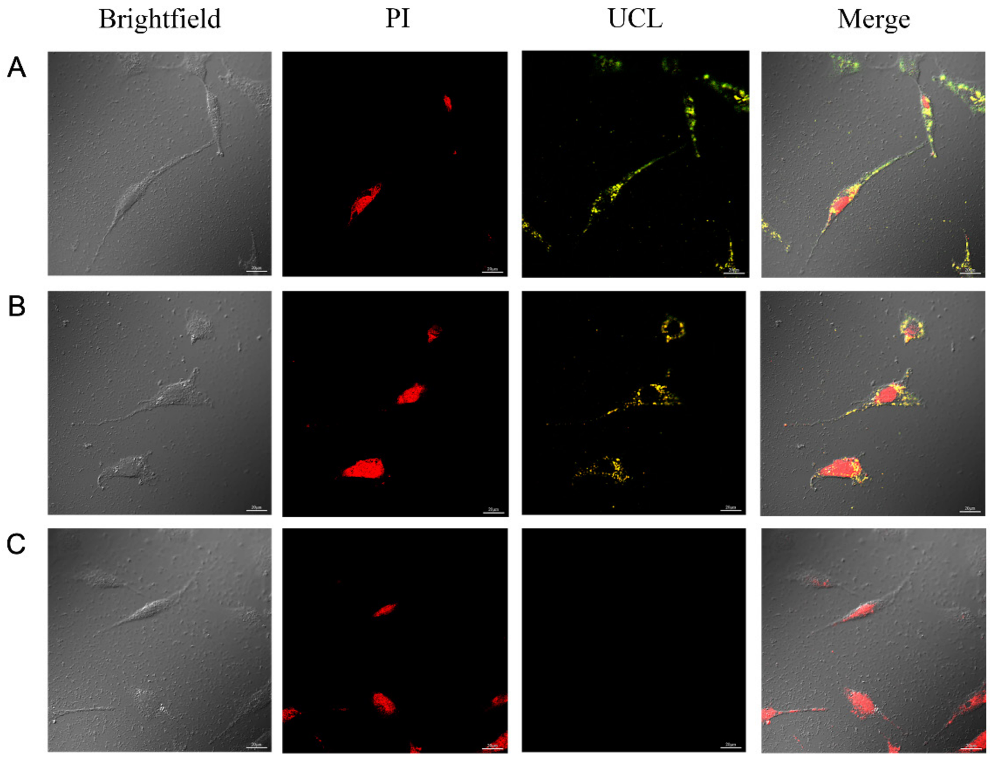

Figure S5. LSUCLM images of MC3T3-E1 cells incubated with UCHRT (A, 200 μg/mL), csUCNP@MSN (B, 200 μg/mL) or PBS (C) for 3 h and then cells were fixed and stained with PI. UCL signals were collected from 500 to 560 nm and 600 to 700 nm. PI signals were collected from 600 to 650 nm.

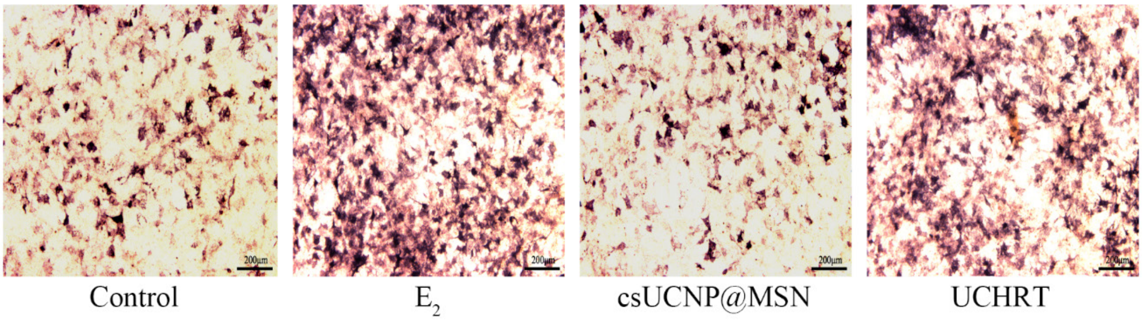

Figure S6. ALP staining of MC3T3-E1 cells after 7 days of treatment. The deeper the staining, the greater the amount of expression.

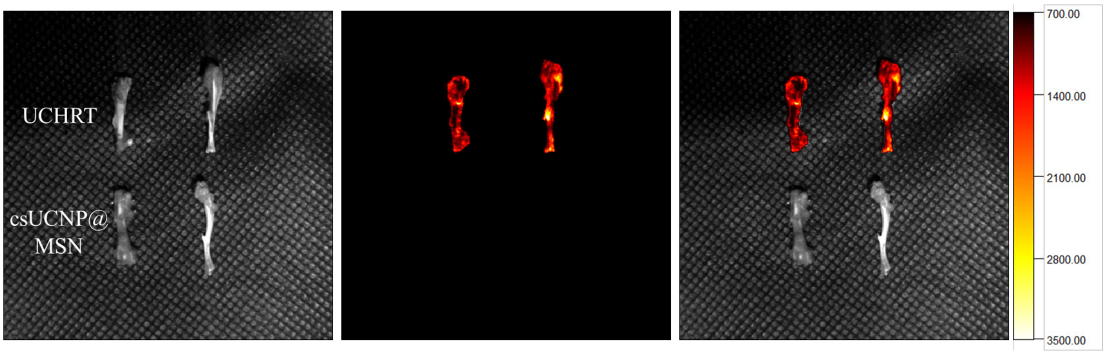

Figure S7. Ex vivo UCL imaging of the mouse bones after UCHRT and csUCNP@MSN aqueous dispersion immersion.

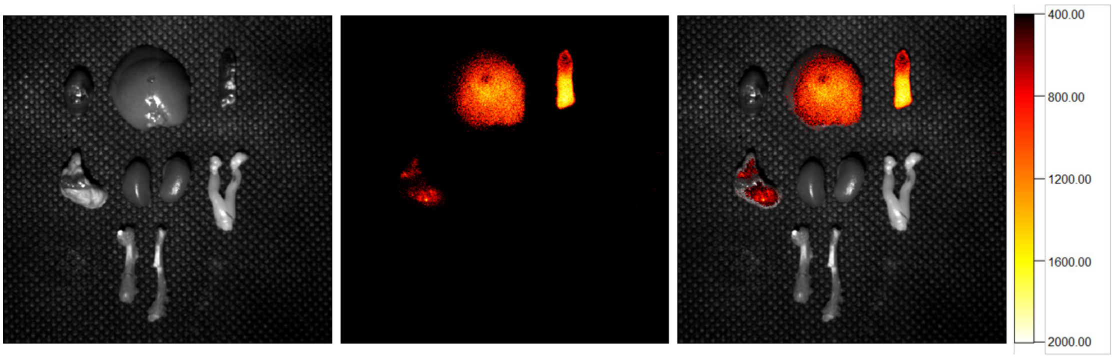

Figure S8. Ex vivo UCL imaging of the mouse organs after injection of

csUCNP@MSN.

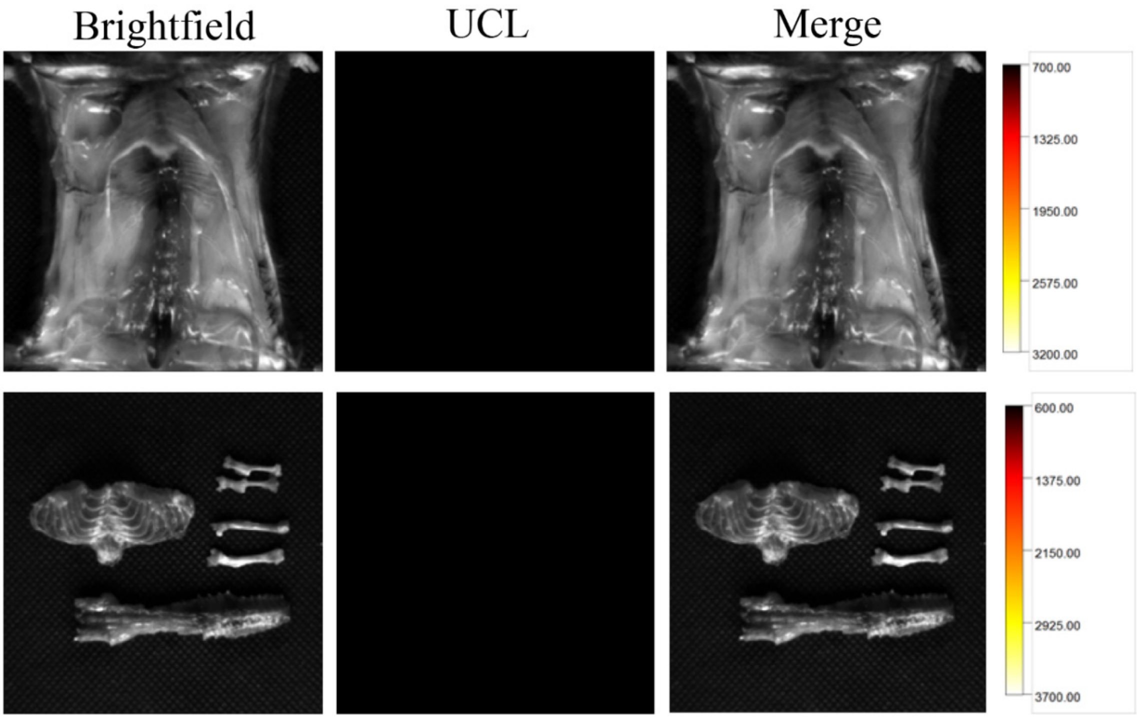

Figure S9. In situ and ex vivo UCL imaging of the mice 6 h after intravenous injection of 200 μL PBS.

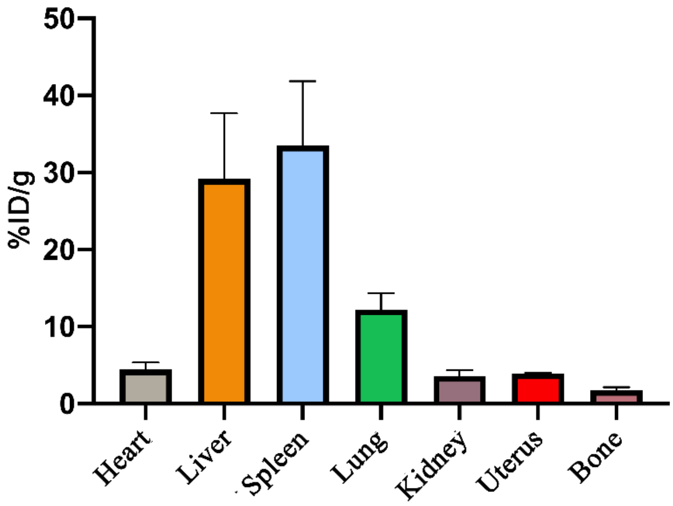

Figure S10. Quantitative distribution of csUCNP@MSN in mice after injection by

inductively coupled plasma atomic emission spectroscopy (ICP-AES).

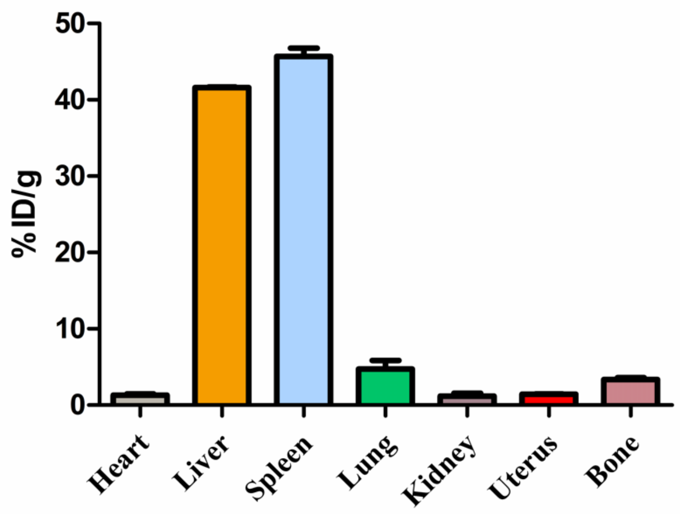

Figure S11. Quantitative distribution of UCHRT in mice after injection by ICP-AES.

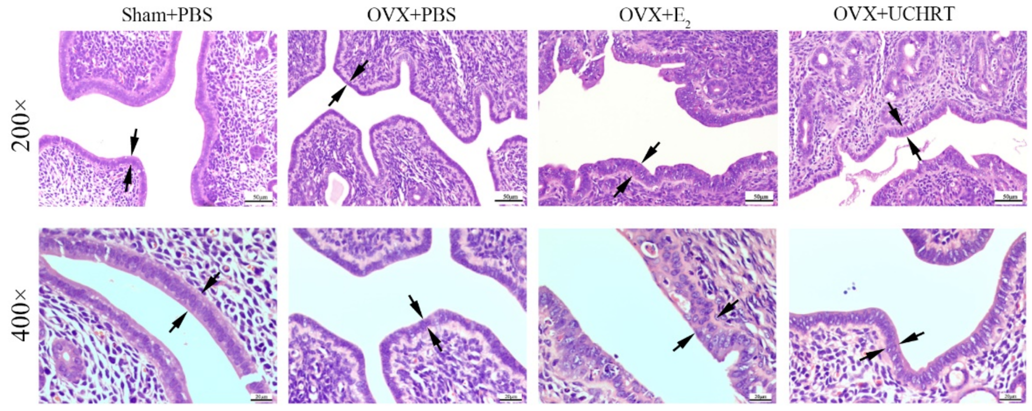

Figure S12. Representative images of effect of UCHRT on uterus histomorphometric after H&E staining (200X, 400X). Luminal epithelium was shown as the black arrow.
